# SAT, a flexible and optimized Web application for SSR marker development

**DOI:** 10.1186/1471-2105-8-465

**Published:** 2007-11-29

**Authors:** Alexis Dereeper, Xavier Argout, Claire Billot, Jean-François Rami, Manuel Ruiz

**Affiliations:** 1 CIRAD, UMR DAP, TA A-96/03, Avenue Agropolis, Montpellier, France

## Abstract

**Background:**

Simple Sequence Repeats (SSRs), or microsatellites, are among the most powerful genetic markers known. A common method for the development of SSR markers is the construction of genomic DNA libraries enriched for SSR sequences, followed by DNA sequencing. However, designing optimal SSR markers from bulk sequence data is a laborious and time-consuming process.

**Results:**

SAT (SSR Analysis Tool) is a user-friendly Web application developed to minimize tedious manual operations and reduce errors. This tool facilitates the integration, analysis and display of sequence data from SSR-enriched libraries.

SAT is designed to successively perform base calling and quality evaluation of chromatograms, eliminate cloning vector, adaptors and low quality sequences, detect chimera or partially digested sequences, search for SSR motifs, cluster and assemble the redundant sequences, and design SSR primer pairs. An additional virtual PCR step establishes primer specificity. Users may modify the different parameters of each step of the SAT analysis.

Although certain steps are compulsory, such as SSR motifs search and sequence assembly, users do not have to run the entire pipeline, and they can choose selectively which steps to perform. A database allows users to store and query results, and to redo individual steps of the workflow.

**Conclusion:**

The SAT Web application is available at , and a standalone command-line version is also freely downloadable. Users must send an email to the SAT administrator tropgene@cirad.fr to request a login and password.

## Background

Simple Sequence Repeats (SSRs) have been shown to be one of the most powerful genetic markers known [1]. They are abundant, having been reported to occur approximately every 6 kb in plant genomes [2], and exhibit a high degree of polymorphism, due to a high mutation rate, which leads to variations in the number of repeat units [3]. Their abundance and hypervariability make SSRs excellent markers for genotype identification, construction of genetic maps, analysis of genetic diversity, and marker-assisted selection of crop plants.

SSR loci are commonly identified by sequencing genomic DNA libraries enriched for SSR sequences [4]. Raw results of this sequencing step usually consist of chromatogram or trace files. The manual analysis of these abundant sequence data, from base calling to the production of specific SSR primer pairs, is laborious and time-consuming.

An automated SSR Analysis Tool (SAT) has been developed to collect sequence information and facilitate the design of PCR primers that will amplify SSRs and their flanking sequences. This software integrates various external tools like Phred, Lucy, d2-cluster, Phrap, CAP3 and ePrimer3. SAT is able to successively perform base calling and quality evaluation of chromatogram files, eliminate cloning vector and low quality sequences, search for SSR motifs, cut chimeric sequences or partial restriction products, cluster and assemble the redundant sequences (forward and reverse products, as well as duplicates), and finally design SSR primer pairs and check primer specificity. An integrated Web interface allows the remote use of this tool.

## Implementation

SAT is composed of three major components: a set of pipelined programs, a relational database and a Web interface. The SAT pipeline consists of PERL modules wrapping different external software programs. In some cases, we reused BioPERL modules like Bio::Tools::RestrictionEnzyme [5]. The SAT database system uses MySQL [6]. The SAT Web interface is implemented with static HTML pages and dynamic PERL/CGI scripts. The server is currently running on a machine with Red Hat LINUX 7 and Apache version 1.3.33.

### Pipeline Components

**Figure 1:**
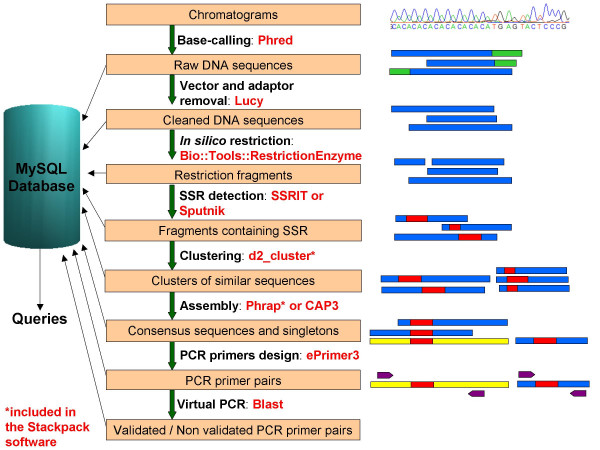
**The SAT workflow**. The SAT workflow consists of eight steps: a. Base calling, b. Cleansing, c. *In silico *restriction digestion, d. SSR detection, e. Clustering, f. Assembly, g. Primer design, h. Virtual PCR. Results of each analysis step are stored in a MySQL database.

The automated process consists of eight steps (Figure 1):

#### Base calling

SAT accepts chromatogram files, in SCF or ABI format, and uses the Phred program [7] to perform base calling and to assign corresponding quality scores. The output of this step is a multi-FASTA sequence file and the corresponding quality file.

#### Cleansing

The pipeline uses the Lucy software [8] to clean the raw sequences by removing low quality sequences and those of the cloning vector and adaptors. The user must specify the quality thresholds and the vector information (vector sequence and vector cloning site). Lucy reads the quality information produced by the Phred program in order to keep good quality regions, i.e. regions within sequences that have higher quality values than a threshold provided by the user. Lucy then compares input sequence with the vector and splice site sequences and adds this clipping information in the header of the initial multi-FASTA file. Sequence and quality files are then clipped by SAT.

#### In silico restriction

SAT performs a virtual restriction to detect chimera or partially digested sequences that may have formed during the SSR enriched library construction. The virtual restriction is performed using the BioPERL module Bio::Tools::RestrictionEnzyme [5]. The user has to specify the restriction enzyme used for library construction.

If the in silico restriction of a specific sequence, using the same restriction enzyme as the one used for the library construction, leads to more than one fragments, this means that the fragment was either a chimeric sequence or a partially digested sequence.

Obviously, chimeric sequences can not be a priori distinguished from partially digested sequences, and we choose then to keep, after the restriction step, all the fragments containing a SSR motif. These fragments would correspond to what one should obtain with a complete digestion and no chimeric sequences.

#### SSR detection

SAT searches for SSRs and keeps the sequences containing an SSR motif. Users may choose between two different programs to perform the SSR search: the SSRIT program [1], which allows the choice of the minimal number of repeats for each pattern of di, tri or tetranucleotide; or the Sputnik software [9], which is offered as an alternative because it presents the advantage of reporting imperfect SSRs.

#### Clustering

SAT first masks the SSR repeat regions before it clusters sequences using the d2_cluster program [10]. Sequences in the same cluster may correspond to either the forward and reverse sequencing of the same locus, the double picking of bacteria during the library construction, or the real repetition of the SSR locus in the genome. Microsatellite sequences are then unmasked for the assembly step.

#### Assembly

Sequences in each cluster are assembled so that consensus sequences are generated. The assembly module uses either Phrap [11] or CAP3 [12], depending on the user's preference. The Phrap assembly is followed by an additional alignment analysis performed by Craw. Whereas Phrap tends to produce the longer contigs, CAP3 is likely to produce fewer errors in consensus sequences [12]. SAT users can select the assembly tool best suited to their needs.

#### Primer design

A new SSR detection step is performed, within each consensus sequence, prior to the PCR primer design stage. The ePrimer3 software [13] then analyzes the flanking DNA sequences in order to define suitable forward and reverse PCR primers with which to assay the SSR loci. Primers are designed with a user-defined set of constraints, such as oligonucleotide melting temperature, primer length, GC content, PCR product size, and positional constraints around the SSR. When a consensus sequence contains several SSRs, the program targets each of them to define local primer pairs, before targeting the whole SSR region to design global primer pairs.

#### Virtual PCR

The virtual PCR step verifies the specificity of the detected PCR primers. This step validates the efficiency of clustering: if primers designed on a specific contig amplify at least another contig, this either means that these two contigs should have been assembled together (thus revealing bad assembly parameters), or that primers have been designed in a conserved region of two distinct loci.

Each set of primer pairs is analyzed for sequence similarity using BLAST [14] against all the sequences (consensus and singletons) of the project.

Using the outputs of the virtual PCR step, SAT classifies sequences in three categories according to the specificity of primers:

a) Sequences for which the primers are specific. Neither the forward nor reverse primer matches other sequences of the project with more than 80% identity. In this case, the first couple of primers found by ePrimer3 are automatically flagged as "validated". If several SSRs are found in the same sequence, the primer pair designed to amplify the whole SSR region is validated. If no global primer is found, primer pairs specific for each SSR are validated.

b) Sequences for which the primers are not specific. At least one primer, among the various primer pairs proposed, matches one or more other sequence of the project with a greater than 80% identity. SAT then uses the Graph9 program [15] to automatically perform a new clustering of these sequences, consensus or singletons, linked by at least one primer. Users will be allowed to restart the sequence alignment with CAP3 and decide whether or not to keep the newly formed consensus sequence by way of an automatic database update.

c) Sequences for which no primer is found because of the constraints applied on ePrimer3. For this class of sequences, user will be also invited to refine results, by running ePrimer3 again, with less stringent parameters to allow the program to design primers.

## Results and discussion

### Web Application

A comprehensive Help page includes details on how to configure and run the pipeline, and how to search, display and reanalyse SAT results.

Users log onto the SAT Web site and choose between running the pipeline or querying the database. In the pipeline entry, users may submit a new project and execute the pipeline. In the database entry, users can consult the analysis results of previous projects, as well as refine the analyses on the consensus sequences and their respective primers.

### Running the Pipeline

#### Pipeline Flexibility

The sequences to be analysed can be provided either as trace files (ABI or SCF format) or as sequence files (FASTA format); therefore SAT can use sequences obtained from different sources (e.g. SSR enriched libraries, BAC sequences, etc.).

**Figure 2:**
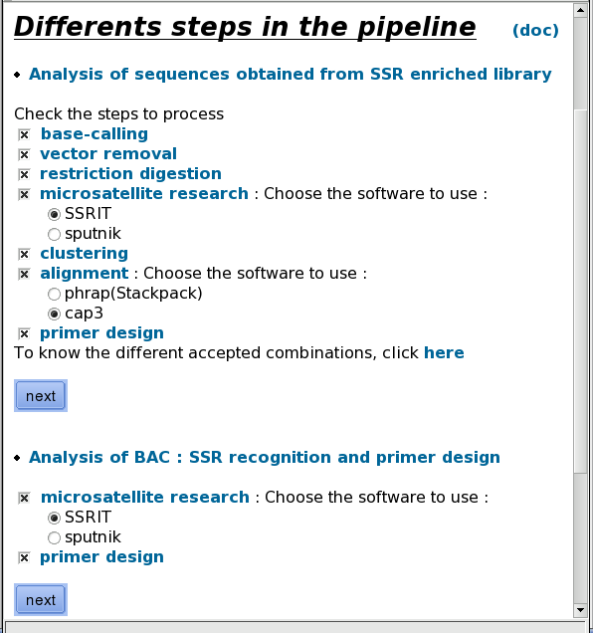
**Pipeline Steps**. Users can easily select which steps of the pipeline to perform. If the base calling step is selected, the user would need to submit an archive or ZIP file containing the chromatograms for analysis. If this step is skipped, a multi-FASTA file containing the nucleotide sequences, with or without the corresponding quality file, would need to be entered. The "Analysis of BAC" option allows FASTA nucleotide sequences to be quickly analyzed in order to detect SSR motifs and design primers, skipping the optional steps of a classical SAT analysis.

The pipeline is flexible enough to allow users to select which of the steps should be launched (Figure 2). If the base calling step is selected, the user would need to submit an archive or ZIP file containing the chromatograms for analysis. If this step is skipped, a multi-FASTA file containing the nucleotide sequences and the corresponding quality file would need to be entered. Submitting a multi-FASTA file is the only option available if the program is to be run from a point after the cleansing step. The virtual restriction digestion and primer design steps may be skipped, but not the SSR discovery and assembly steps, which are the core of the pipeline. The clustering step may only be skipped when CAP3 is selected.

Although SAT has been optimized for the analysis of sequences obtained from SSR-enriched libraries, the "Analysis of BAC" option (Figure 2) allows FASTA nucleotide sequences to be quickly analyzed in order to detect SSR motifs and design primers, skipping the optional steps of a classical SAT analysis.

By default, the maximum number of sequences accepted by SAT is limited to 6000 chromatograms/sequences, but upon special request from users, this limit can be augmented.

#### Setting parameters and pipeline execution

**Figure 3:**
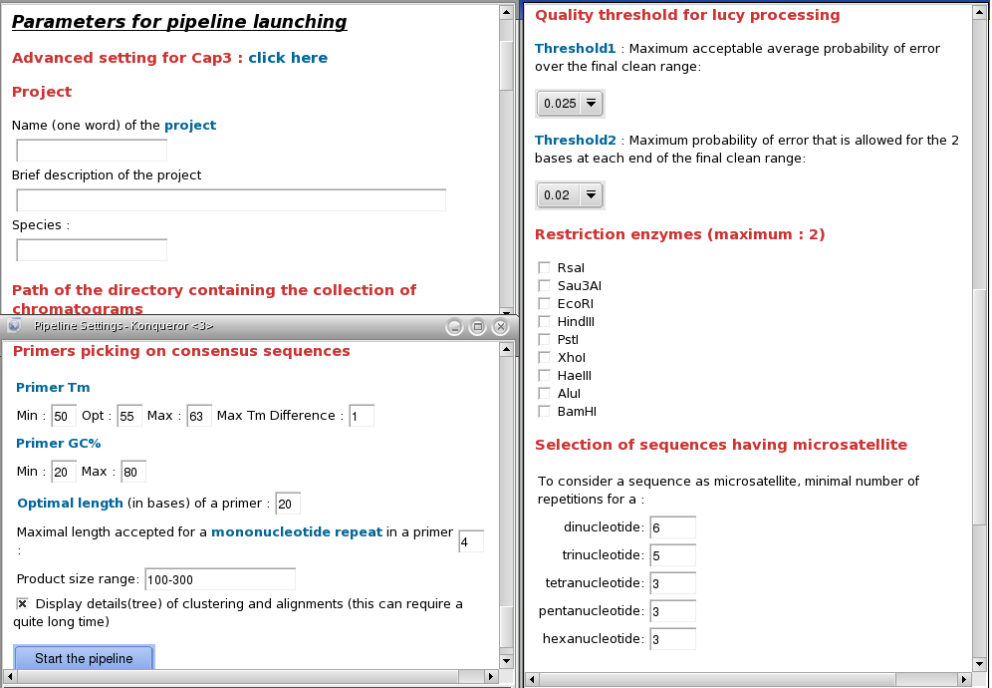
**Parameters setting**. Users are provided with options to set the parameters for each of the different steps in the pipeline. At the end of the analysis, users may choose to re-run the pipeline with modified parameters.

Users have the option to set the parameters for each of the different steps along the pipeline (Figure 3). When the pipeline is launched, a new HTML page presents the results progressively while the sequences are analyzed. At the end of the process, users have access to the detailed reports of all the steps in the pipeline. They may save their results into the database, download a summary report (as an Excel file) or re-run the pipeline with modified parameters. When users choose to import and save the results into the database, the virtual PCR analysis is launched automatically.

### Querying the Database

The pipeline is linked to a database, which allows users to store and query results, as well as refine some of the analyses, including sequence assembly, new primer design for sequences without primers and the manual validation of primer pairs.

#### Primers Pairs Validation

A "validated" status is automatically assigned to the first pair of primers that are found to be specific for either a consensus or singleton sequence. However, users may modify the "validated" status after checking the SAT results. Information on the validated primers can be exported.

#### Project management

SAT is equipped with password protection so that researchers can only access their own project and share it with collaborators. New users must send an email to the SAT administrator tropgene@cirad.fr to request a login and password.

For each project, users may visualize the analysis results (Project Description, Summary and Validated Primers tabs), refine the analysis on the consensus sequences and their respective primers (Sequences Analysis tab), submit personal primer pairs (Import Data tab) and download the different results (Export Data tab).

#### Results visualization

**Figure 4:**
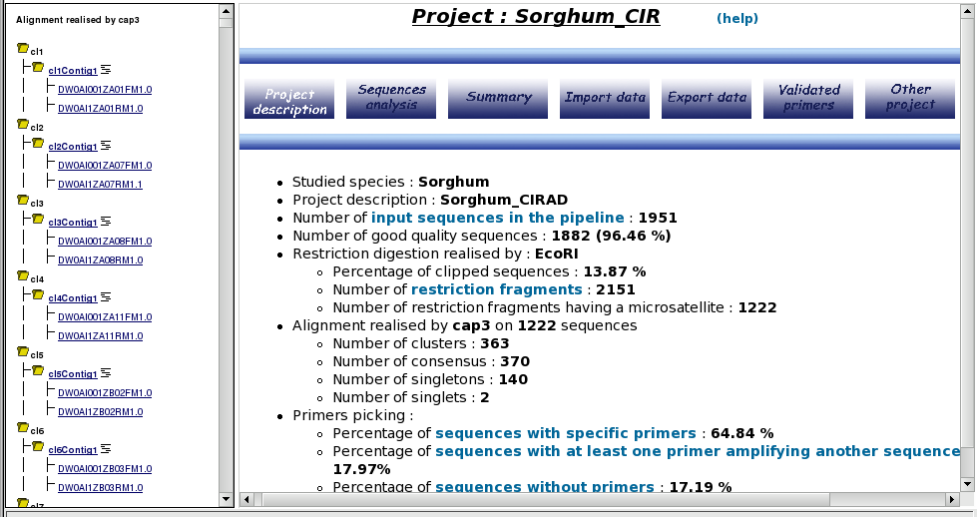
**Overview of the project results**. A treeview display on the left-hand side of the screen provides a clearly-visible distinction between the different sequence assemblies (contigs, singleton and singlet) that were generated by the clustering and alignment steps The Project Description tab allows the user to consult statistics on the different analysis steps, with links to the corresponding outputs.

A treeview display on the left-hand side of the screen provides a clearly-visible distinction between the different sequence assemblies (contigs, singleton and singlet) that were generated by the clustering and alignment steps. Singletons are defined as unique sequences that could not be assembled in a cluster whereas singlets are unique sequences that were assembled in a cluster but could not be assembled in a contig. Each leaf of the tree corresponds to a sequence identifier (Figure 4). Clicking on an identifier link opens a new window displaying a colored representation of the sequence, highlighting the positions of the different SSR motifs and the associated primer pairs.

The Project Description tab allows users to consult statistics on the different analysis steps, with links to the corresponding outputs (Figure 4).

The Summary tab provides general information on the SSRs identified and the candidate PCR primers. This table includes the consensus identifiers (ID) and corresponding sequence lengths, the cluster and contig ID, the original sequence ID derived from the initial FASTA headers or chromatogram names, links to the alignments for each contig, SSR motifs, SSR sizes, SSR start and end positions, forward and reverse sequences of the set of candidate primers, and the forward and reverse sequences of the validated primers, including the corresponding PCR product sizes.

The Validated Primers tab displays the list of validated primers with specific details: Sequence ID, SSR motif, SSR start and stop positions, ID, sequence, melting temperature, sequence size (for forward and reverse primers respectively) and the final PCR product size. This list may be downloaded as an Excel file.

#### Sequence Analysis

A powerful feature of SAT is the possibility for users to check and refine the analysis on the consensus sequences and their respective primer pairs.

**Figure 5:**
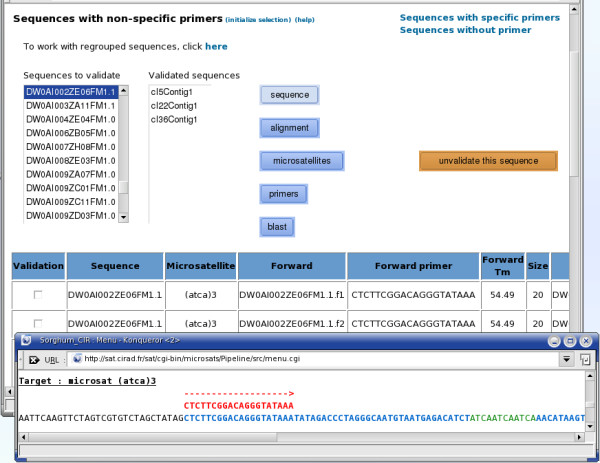
**Sequences Analysis menu**. This displays the nucleotide sequence, the alignment of the consensus sequences, the coloured representation of the SSR motifs and the set of candidate primer pairs, for each sequence of the project. Users can perform a BlastN of a sequence against the NCBI nucleotide sequence databank and can also manually modify the "validated" status for the different sets of primer pairs.

**Figure 6:**
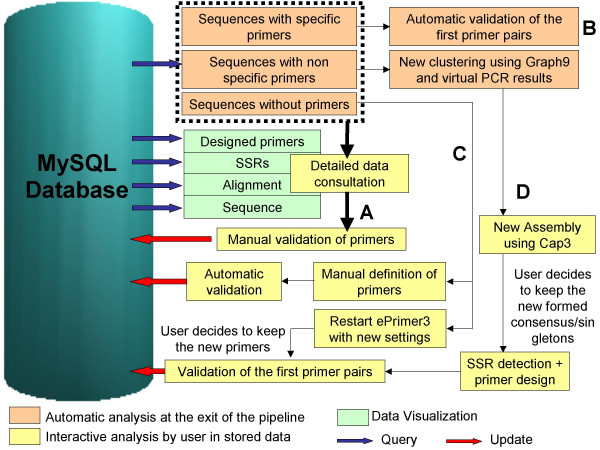
**Workflow for refining the SAT analyses on the consensus sequences and their respective primers pairs**. The user can view the full details (Primers, SSRs, Alignment, Sequence) for each sequence and choose to manually validate the corresponding primer pairs (A). For sequences with "specific" primers, the first pair of primers found by ePrimer3 automatically get the "validated" status (B). For sequences without primers, SAT proposes to run ePrimer3 again, using modified analysis parameters, and the user decides whether or not to accept any newly detected primers into the database. Alternatively, a primer pair for each remaining sequence can be entered manually (C). For sequences with non-specific primers, SAT proposes to restart the sequence alignment using CAP3, within each subgroup redefined by the results of Blast and Graph9, with the option of keeping the newly formed consensus sequence (D).

The Sequences Analysis tab (Figure 5) displays the nucleotide sequence, the alignment of the consensus sequences, the coloured representation of the SSR motifs and the set of candidate primer pairs, for each sequence of the project. Users can perform a BlastN of a sequence against the NCBI nucleotide sequence databank and can also modify the validation status of different sets of primer pairs (Figure 6A).

For sequences with "specific" primers, the first couple of primers found by ePrimer3 automatically gets a "validated" status (Figure 6B).

For sequences without primers, SAT offers to run ePrimer3 again using modified analysis parameters. The user then decides whether or not to accept any newly detected primers into the database, which will automatically validates them.

For any remaining sequences without detected primers, a table shows statistics on the main reasons for the failure of ePrimer3 for each sequence (e.g. low Tm, primer found in excluded region) and another table presents the global incidence of each reason. Users are also given the opportunity to propose a primer pair for each remaining sequence and save it in the database (Figure 6C).

For sequences with non-specific primers, SAT proposes to restart the sequence alignment (within each subgroup redefined by the results of Blast and Graph9) using CAP3, with the option of keeping the newly formed consensus sequence (Figure 6D).

#### Import Data

SAT allows existing primer pairs to be imported and blasted against all the initial or final sequences (consensus and singletons) of the project. If an exact match is found for the forward and reverse primer sequences, they are automatically registered in the SAT database. Imported primers are given priority over others, so any that may have already been validated for the same sequence are invalidated. Details of the analysis (blast outputs, amplified sequences and the association between validated primers and targeted sequences) of the submitted primers are also available.

#### Export Data

The Export Data tab allows users to download a multi-FASTA file containing the different members of a particular cluster or contig, a multi-FASTA file compiling all the final consensus and singletons, as well as a multi-FASTA file containing all the primers sequences. An Excel file listing all the validated primer pairs and a summary text file of the SAT analysis are also available.

#### Downloadable version

A standalone command line version of the program is freely available through the download link on the SAT home page.

This package, which runs on LINUX platforms, contains all the PERL scripts and modules needed for running the pipeline. The SAT application is easily extendable; data marshalling is used to transfer data between each PERL program, so a developer can easily add new PERL modules.

### Benefits

Previous software packages have been implemented to automatically discover SSR markers, but their functionality is more limited when compared to SAT. SSR Primer Discovery Tool [16,17] only uses FASTA formatted data as input and is not able to start analysis from base-calling. Moreover, only a few constraints in the step of PCR primer design may be defined by the user. MicrosatDesign [18] can accept sequencer trace files, but may only be used in command line mode on UNIX systems, without an interactive interface and database for project management. Thiel et al. [19], and more recently Martins et al. [20] proposed a suite of software to improve the determination of microsatellite markers, but to our knowledge, SAT is the first integrated Web application optimized for automatic development of SSR markers from chromatogram files.

SAT is highly flexible, so as to allow users to create their own SSR analysis workflow. Either chromatogram files or FASTA formatted sequences can be submitted to the program. In the case of chromatogram inputs, the initial evaluation of sequence quality is important in avoiding errors in the sequence assembly step that follows, allowing suitable PCR primers to be identified with confidence. Users may selectively choose which steps of the SAT pipeline to perform and have the option to modify the parameters at each stage of the SAT analysis. In the case of the PCR primer design step, the default parameters are based on our empirical data. However, users are encouraged to adjust them and find combinations appropriate for their situation. The modular architecture of the SAT pipeline renders this system easily extensible, which will facilitate the addition of new features and programs according to user feedback.

SAT was conceived to optimize the design of good quality SSR markers. For example, the in silico restriction digestion will detect potential chimeric sequences. The Sequences Analysis step permits users to refine the analysis at different levels: sequence clustering and assembly, primer detection and primer specificity. To facilitate easy data exploration, SAT displays Web-based results, which may be accessed through the internet. The project management database of SAT allows users to store results, to query all the intermediate results, and to repeat some of the steps. SAT presents detailed and well organized information, so that scientists can focus on the biological aspect of their projects, saving time from routine data processing work.

In our laboratory, the entire SAT pipeline was successfully used on different SSR enriched banks (sorghum, coffee, guava, yam, locust, *Amblyomma variegatum*) and EST banks (coffee, citrus) (Table 1). The application has analyzed libraries enriched for SSRs with more than 2700 sequences (sorghum). The "Analysis of BAC" option of SAT was used on 21 *Musa *BAC sequences of 60 kb to 180 kb. An example of experimental validation of the SAT application was the development of new markers from the SSR sorghum enriched library (Table 1). Among the 346 primer pairs flanking SSR loci defined by SAT, a subset (132) were selected and used for screening polymorphisms among 15 sorghum cultivars, including parents of the mapping population sar10 × ssm249. Of the 132 primer pairs, 24 (18%) were found monomorphic between the 15 sorghum cultivars examined, and for 2 primer pairs (1.5%) no amplification was detected. Twelve of the validated new SSR markers have been integrated into the sorghum SSR Kit to study sorghum genetic diversity [21]. This SSR Kit has been elaborated in the frame of a large international genotyping project (3400 sorghum accessions × 48 SSR loci) supported by the Generation Challenge Program [22].

**Table 1:** Examples of SAT results

**Origin of sequences**	**Species**	**Number of sequences analyzed**	**Number of sequences with SSRs**	**Consensus**	**Singletons and singlets**	**Sequences with specific primer pairs**	**Sequences with non specific primer pairs**	**Sequences without primers**
**SSR enriched banks**	**Sorghum**	2747	1176	379	144	346 (66.16%)	63 (12.04%)	114 (21.80%)
	**Coffee**	1149	948	246	251	295 (59.36%)	45 (9.05%)	157 (31.59%)
	**Guava**	464	440	57	272	190 (57.75%)	6 (1.82%)	133 (40.43%)
	**Yam**	209	172	14	121	54 (40.00%)	12 (8.89%)	69 (51.11%)
	**Locust**	182	171	8	143	54 (35.76%)	16 (10.60%)	81 (53.64%)
	**Amblyomma variegatum**	86	79	8	38	10 (21.74%)	15 (32.61%)	21 (45.65%)
**EST banks**	**Coffee**	2529	228	3	219	176 (79.28%)	13 (5.86%)	33 (14.86%)
	**Citrus**	1842	350	36	270	213 (69.61%)	44 (14.38%)	49 (16.01%)

## Conclusion

SAT, SSR Analysis Tool, is a flexible and user-friendly Web application, optimized for SSR marker identification and the design of specific PCR primers. The major advantages of SAT are that it saves time and reduces the errors that might be introduced by analyzing sequences by hand; users only need to upload their sequences or chromatograms, and select the parameters appropriate to the tools.

In addition to its utility in analyzing SSR-enriched libraries, SAT can use any type of sequence (EST, mitochondrial genome, etc.) to detect SSRs and design the necessary primers. This tool is generic enough to be used on the DNA of any organism.

## Availability and requirements

• Project name: SAT, SSR Analysis Tool

• Project home page: 

• Operating system: Web access, standalone: UNIX/LINUX

• Programming language: PERL

• Licence: GNU GPL, free for academic and non-academic users

• Any restrictions to use by non-academics: none

## Authors' contributions

AD wrote all the application. XA helped to design the software architecture. JFR and CB instigated and coordinated the project. MR provided advice and guidance throughout the project and drafted the manuscript. All authors read and approved the final manuscript.
